# RETREAT Score Accurately Predicts the Long-Term Risk of HCC Recurrence After Liver Transplantation: A Single-Center Real-Life Validation

**DOI:** 10.3390/cancers18040556

**Published:** 2026-02-09

**Authors:** Flavia Neri, Mauro Viganò, Stefania Camagni, Marco Fabrizio Zambelli, Alessandro Loglio, Massimo De Giorgio, Riccardo Muglia, Lisa Licini, Andrea Francavilla, Paolo Marra, Sandro Sironi, Paola Anna Erba, Irene Gotuzzo, Andrea Gianatti, Stefano Fagiuoli, Domenico Pinelli

**Affiliations:** 1 Unit of General Surgery 3 and Transplantation, ASST Papa Giovanni XXIII, 24127 Bergamo, Italy; fneri@asst-pg23.it (F.N.); scamagni@asst-pg23.it (S.C.); marcozambelli@asst-pg23.it (M.F.Z.); dpinelli@asst-pg23.it (D.P.); 2 Gastroenterology, Hepatology and Transplantation Unit, ASST Papa Giovanni XXIII, 24127 Bergamo, Italy; aloglio@asst-pg23.it (A.L.); mdegiorgio@asst-pg23.it (M.D.G.); sfagiuoli@asst-pg23.it (S.F.); 3 Department of Medicine and Surgery, University of Milano-Bicocca, 20126 Milan, Italy; pmarra@asst-pg23.it (P.M.); ssironi@asst-pg23.it (S.S.); perba@asst-pg23.it (P.A.E.); 4 Department of Radiology, ASST Papa Giovanni XXIII, 24127 Bergamo, Italy; rmuglia@asst-pg23.it; 5 Unit of Pathology, Department of Oncology and Hematology, ASST Papa Giovanni XXIII, 24127 Bergamo, Italy; llicini@asst-pg23.it (L.L.); agianatti@asst-pg23.it (A.G.); 6 FROM, Fondazione per la Ricerca Ospedale di Bergamo ETS, 24129 Bergamo, Italy; afrancavilla@fondazionefrom.it; 7 Department of Nuclear Medicine, ASST Papa Giovanni XXIII, 24127 Bergamo, Italy; igotuzzo@asst-pg23.it

**Keywords:** HCC, recurrence, survival, liver transplantation, RETREAT score

## Abstract

**Background:** Liver transplantation (LT) for Hepatocellular carcinoma (HCC) is still burdened by a significant risk of tumor recurrence. The aim of this study was to apply the Risk Estimation of Tumor Recurrence After Transplant (RETREAT) score for prediction of HCC recurrence and survival. **Methods**: This retrospective study included all adult patients who consecutively underwent LT for HCC from January 2000 to July 2022 at a single institution. **Results**: During a median follow-up of 64 months, 56 (19%) out of 298 patients [54%, 36% and 10% with a RETREAT score equal to 0/1 (low), 2/3 (medium) and 4–6 (high), respectively] experienced HCC recurrence after a median of 31 months. The HCC recurrence rates at 1, 5 and 10 years were respectively 4%, 16% and 23%. The 5-year RFS for low-, medium- and high-risk groups were 93%, 78% and 58% (*p* < 0.001), respectively. In the competitive risk analysis, the medium- and high-risk RETREAT groups had respectively a 2.3 HR (*p* = 0.017) and 6.4 HR (*p* < 0.001) of HCC recurrence compared with the low-risk group. Overall, 119 patients died [32 (27%) due to HCC recurrence], and the 5-year survival was 74% (95%, 86% and 61% for low-, medium- and high-risk groups, respectively). In the multivariate analysis, the RETREAT score was associated with the overall survival only for the highest risk class, which yielded a 2.5 HR of death compared with the lowest risk categories (*p* < 0.001). **Conclusions**: This study validates the RETREAT score and confirms its ability to predict the risk of HCC recurrence.

## Simple Summary

Hepatocellular carcinoma (HCC) is one of the main indications for liver transplantation (LT). Despite rigorous selection criteria, the risk of HCC recurrence, although low, remains present. Over the years, various models have been developed to improve the ability to predict recurrence, and among these, the RETREAT (Risk Estimation of Tumor Recurrence After Transplant) score is one of the most validated. However, in recent years, LT eligibility criteria for HCC have expanded, as has the increased use of down-staging strategies, thus increasing the heterogeneity of patients considered for LT. In this evolving landscape, predicting tumor recurrence and survival has become even more essential, particularly with regard to the intensity of surveillance and the choice and dosage of immunosuppression in patients at high risk of recurrence. Our retrospective study on a large cohort of patients undergoing LT for HCC confirmed the ability of RETREAT to predict the risk of HCC recurrence.

## 1. Introduction

Hepatocellular carcinoma (HCC) is one of the leading indications for liver transplantation (LT) worldwide, offering the unique advantage of simultaneously removing both the tumor and the underlying liver disease. However, post-transplant HCC recurrence remains a major determinant of long-term mortality, occurring in approximately 10–20% of patients despite adherence to established selection criteria [1,2,3]. Historically, patient selection for LT has relied primarily on radiological criteria—most notably as the Milan Criteria (MC)—which demonstrated that limiting tumor size and number could significantly reduce recurrence and provide excellent long-term outcomes with a 5-year survival rate exceeding 70% [4]. Nevertheless, subsequent studies have highlighted the heterogeneity of tumor biology within these criteria, with a non-negligible risk of recurrence even among patients on MC, and, conversely, favorable outcomes in selected patients beyond them [5,6]. This has led to a paradigm shift from purely morphological assessment toward integrated models incorporating biological and dynamic tumor features. Several factors have been consistently associated with post-LT HCC recurrence, including underlying liver disease, radiological features and response to bridge or down-staging treatment, microvascular invasion (MVI), poor tumor differentiation, elevated alpha-fetoprotein (AFP) levels and tumor burden at explant pathology [1,7,8,9,10,11,12,13]. Among these, AFP has emerged as a robust surrogate marker of tumor aggressiveness and is now incorporated into multiple prognostic models and allocation policies [14,15,16]. Importantly enough, explant’s pathology continues to be a significant prognostic feature that was not detectable pre-LT, underscoring the limitations of pre-operative radiological staging alone [10]. Moreover, recent advanced predictive approaches, such as radiomics-based models for MVI assessment, while promising, still exhibit methodological heterogeneity and insufficient accuracy for reliably predicting HCC recurrence in clinical practice [9].

In this context, the RETREAT score (Risk Estimation of Tumor Recurrence After Transplant) developed by Mehta et al. represents one of the most widely validated tools for post-LT risk stratification [17]. This score integrates three objective variables readily available at the time of LT (AFP levels) and at explant examination, i.e., the presence of MVI and the sum of the largest viable tumor diameter plus the number of viable tumors. This model demonstrated excellent discriminatory ability, with 5-year recurrence risks ranging from <5% in patients with a score of 0 to >75% in those with scores of ≥5 [17]. Subsequent external validations across different geographic regions and transplant cohorts have confirmed the robust predictive performance of this score, supporting its clinical utility for identifying patients at low vs. high risk of tumor recurrence [18,19,20,21].

However, in recent years, the progressive expansion of liver transplant eligibility criteria for HCC has significantly increased the heterogeneity of patients considered for LT. While broader morphological thresholds and down-staging strategies have improved access to potentially curative therapy, they have also heightened concerns regarding the accuracy of pre-operative radiological staging, post-LT tumor recurrence and long-term survival. In this evolving landscape, prediction of HCC recurrence and survival has become essential, particularly regarding surveillance intensity, choice and dosages of immunosuppression, not to mention the intriguing and futuristic possibility of adjuvant therapy in patients who are found to be at high risk of tumor recurrence.

The aims of this study were to evaluate the rate of tumor recurrence in patients who underwent an LT for HCC and to validate the accuracy of the RETREAT score in stratifying both the recurrence and the overall survival in a real-world European setting, different from the original one on which the score was based.

## 2. Materials and Methods

### 2.1. Study Design

This single-center retrospective cohort study included all consecutive patients who underwent LT with HCC between January 2000 and July 2022 at a single Liver Transplant Center in Northern Italy. Patients were excluded from this study in case of

(1) Early in-hospital post-LT death.
(2) Explant pathology showing no evidence of HCC in patients who had not received locoregional therapies (LRTs); cholangiocarcinoma or mixed hepatocellular cholangiocarcinoma.
(3) Insufficient data to calculate the RETREAT score.

The end of follow-up was set in December 2025. All patients provided written informed consent at the time of listing for LT (institutional procedure “PSp01PG3MQ7” for organ transplantation and local ethics committee approval “132-21”). Serum AFP levels were determined by ImmunoAssay in Electrochemistry Luminescence ‘ECLIA’ (Roche Diagnostic GmbH, Mannheim, Germany).

### 2.2. Pre-LT Management of HCC and LT Eligibility

Until 2018, in our Liver Transplant Center, we considered all patients on MC, defined as a single tumor of ≤5 cm or up to three nodules, each of ≤3 cm, in the absence of macrovascular invasion or extrahepatic spread, as suitable for LT [4]. After 2018, the Up-to-Seven Criteria were adopted, supported by data that modest expansion could be oncologically acceptable [13]. Since 2018, the role of LRTs has become central in pre-LT HCC management. Liver resection, trans-arterial chemoembolization (TACE), radiofrequency ablation (RFA) or microwave ablation (MWA), and trans-arterial radioembolization (TARE) were increasingly used to control tumor progression during prolonged waiting times (bridge therapy) but also as a down-staging for all patients initially beyond accepted criteria, i.e., out of the Up-to-Seven Criteria. In the latter cases, if such a procedure was approved, the patients proceeded with down-staging and were subsequently activated in the waiting list if they obtained a post-LT predicted 5-year survival probability of 65% or more, calculated through the Metro-ticket score [13]. In our center, all patients with HCC are routinely discussed within a multidisciplinary tumor board (MTB), which includes interventional radiologists, transplant surgeons, oncologists and transplant hepatologists, to assess transplant eligibility. Patients treated with tyrosine kinase inhibitors (TKIs) before transplantation were also included in this study; since immunotherapy became available after 2022, no patients receiving this treatment were included in this study.

All HCC patients on the waiting list for LT underwent second-level imaging every 3 months. If, at any time, the tumor exceeded MC (before 2018) or the Up-to-Seven Criteria (after 2018), patients were suspended from the waiting list, and the case was re-discussed in the MTB to consider other treatment options.

### 2.3. Post-Transplant Follow-Up

Post-LT follow-up consisted of regular visits at the outpatient clinic, monitoring of AFP, and an abdominal and chest contrast-enhanced CT scan at 3 months, and then every 6 months up to the third post-operative year and annually thereafter. Additional liver MRI or whole-body 18-FDG PET/CT imaging was performed according to clinical judgment. Diagnosis of HCC recurrence was based on radiological and/or histopathological reports.

### 2.4. Milan Criteria and up to Seven Criteria on Explant

All patients were classified as within or beyond the MC or Up-to-Seven Criteria based on the explant pathology report. The RETREAT score was calculated for each patient as previously reported [17]. Patients with an AFP of <20 ng/mL, no MVI in pathology, and completely necrotic tumor(s) on explant after LRTs were given 0 points.

### 2.5. Data Collection and RETREAT Score

The following data were collected from the patients’ clinical charts at the time of LT: sex, age, body mass index (BMI), underling liver disease etiology, Mayo End-Stage Liver Disease (MELD) and Child–Pugh score, number of HCC treatments before LT, radiological features of HCC, waitlist time, serum creatinine, total bilirubin, international normalized ratio (INR) and AFP. Histological examination of the explant native liver was performed by expert pathologists (LL and AG) with the aim of detecting the number of lesions and the dimensions of the largest one, the sum of the diameters (in cm), tumor grade (according to the Edmondson–Steiner grade), and MVI. Based on these data, the RETREAT score was calculated for each patient in comparison with the histological MC and Up-to-Seven Criteria.

For the correlation analysis, the RETREAT score was grouped into three categories:

- Low (0/1);
- Medium (2/3);
- High (4–6).

In the original score proposed by Mehta et al. [17], patients were classified as high-risk when the score was ≥5. However, in the conclusions of the article, different recommendations were suggested for patients with a score of 4 compared with the lower-risk classes, as the risk of HCC recurrence for this group exceeded 20% at 5 years. Therefore, in the present study, a score of ≥4 was also considered indicative of high risk.

HCC recurrence management and any event after LT during follow-up (from the date of LT to the last outpatient visit or the time of exitus) were also collected. The time to HCC recurrence was calculated from the date of LT to the date of first detection of tumor relapse, and patient survival was the period from the LT to the time of death or last follow-up visit.

### 2.6. Statistical Analysis

The cumulative incidence function (CIF) for recurrence was estimated using the time from LT to the event, considering death without recurrence as a competing event.

Statistical analysis was performed with SPSS 19.0 package (SPSS, Chicago, IL, USA) and R (version 4.5.2 http://www.r-project.org). The data are presented as either means (SD) or medians (interquartile range (IQR)) where appropriate. Associations between variables were tested using Student’s *t*-test, chi-square, Pearson correlation, or their nonparametric equivalents, when appropriate. Recurrence probabilities were estimated (at 1, 5, and 10 years) using the Kaplan–Meier method. Recurrence probabilities were compared for patients within and beyond the MC and Up-7-C (based on explant pathology) and across RETREAT scores with log-rank tests. Patients lost to follow-up were censored at the time of the last tumor-free visit. A multivariable Cox model was fitted to adjust the effect of the RETREAT score on HCC recurrence and survival. Hazard ratios (HRs) and corresponding 95% confidence intervals (CIs) were reported. Kaplan–Meier curves were constructed to evaluate the survival of the patients according to the different RETREAT scores. In addition, overall survival probabilities were estimated using the Kaplan–Meier method and compared with the log-rank test for low- and high-risk groups.

## 3. Results

### 3.1. Patients’ Characteristics

We retrospectively enrolled 298 patients. Most of the recipients were male (89%), with a median age of 58 years (IQR: 53–63) and a median BMI of 25.6 (IQR: 23.3–28). The most frequent etiology of liver disease was viral (45% for hepatitis C virus infection, and 24% for hepatitis B virus infection) and alcohol-related (21%). Overall, 269 (90%) patients were cirrhotic with compensated liver disease, as shown by the low median MELD score (11 with an IQR of 8–16). Prior to LT, 255 (86%) patients underwent LRTs as a bridge or down-staging. Overall, the patients underwent several LRTs: nine underwent liver resection, 303 RFA or MWA, 155 TACE, and 11 TARE. The baseline demographic features of the recipients are shown in Table 1.

### 3.2. Pathological Features at Explant and RETREAT Scores

At explant, the pathologist detected a median of two nodules (IQR: 1–3); the greatest had a median diameter of 2.5 cm (IQR: 2–3.5), and the sum of all diameters measured a median of 4 cm (IQR: 2.7–5.5). The median tumor grade was 2 (2–3), and MVI was present on 21% of the native livers. Based on the histological findings, 87% and 66% of the patients were within the Up-to Seven Criteria and MC, respectively. Among the different RETREAT scores, the patients were distributed as follows: 0 (*n* = 4, 1%), 1 (*n* = 157, 53%), 2 (*n* = 52, 17%), 3 (*n* = 56, 19%), 4 (*n* = 16, 5%), 5 (*n* = 9, 3%) and 6 (*n* = 4, 1%). According to the RETREAT score, we grouped the population into three categories of recurrence risk: low (scores 0/1, *n* = 161, 54%), medium (scores 2/3, *n* = 108, 36%) and high (scores 4–6, *n* = 29, 10%), as reported in Table 2.

### 3.3. HCC Recurrence

During a median time of 64 months (IQR: 29.5–116.5) after LT, 56 patients (19%) experienced HCC recurrence after a median of 31 months (IQR: 16.1–82.8) from transplantation. The estimated cumulative rate of HCC recurrence at 1, 5 and 10 years was respectively 4%, 16% and 23%. Forty-two HCC recurrences (75%) occurred within 5 years from LT, 52 (93%) within 10 years and 55 (98%) within 15 years. Most of the recurrences were in the liver (*n* = 17, 30%) and lungs (*n* = 12, 22%), then bone skin (*n* = 2, 3%) and lymph nodes (*n* = 5, 9%), whereas in 19 cases (34%), recurrence occurred at multiple sites simultaneously.

As expected, the HCC-RFS tended to decrease along with the increase in the RETREAT scores, although without a linear correlation. The RFS at 1 and 5 years was 100% for a RETREAT score of 0; 99% and 93% for a score of 1; 94% and 76% for a score of 2; 98% and 79% for a score of 3; 81% and 53% for a score of 4; 89% and 71% for a score of 5; and 75% and 50% for a score of 6. The median RFS was reached only for a score of 6 at 3.2 years.

**Figure 1:**
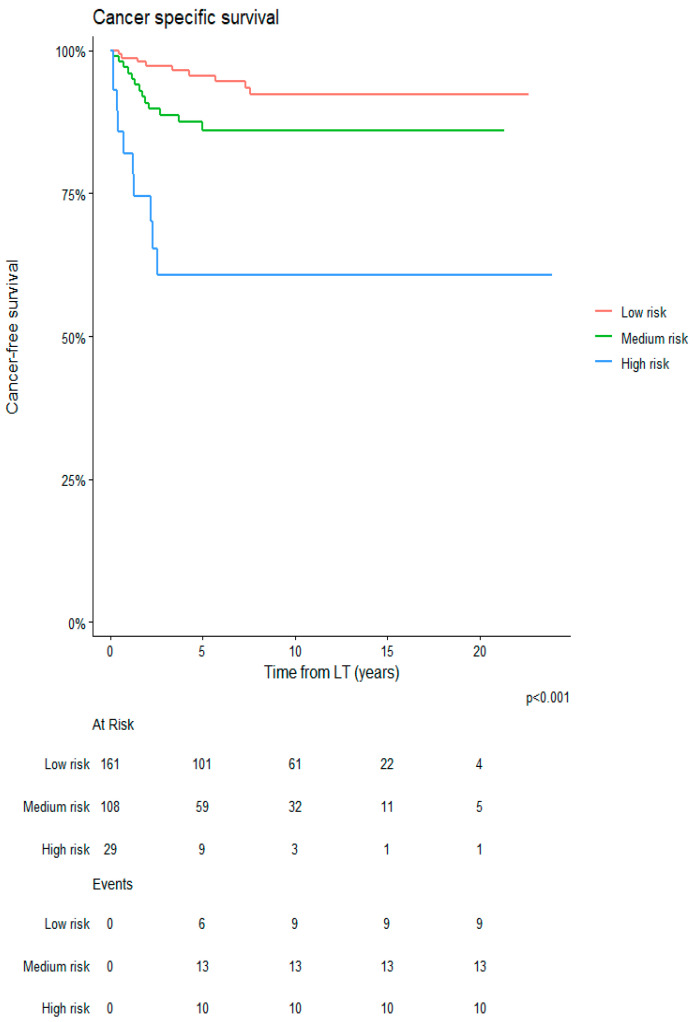
HCC recurrence-free survival according to the categories of the RETREAT score during the study period.

Figure 1 depicts the HCC-RFS according to the categories of the RETREAT score during the entire study period. The HCC-RFS values for low-, medium- and high-risk classes were 99%, 96% and 82% at 1 year and 93%, 78% and 58% at 5 years (*p* < 0.001), respectively. The median RFS was reached at 5.5 years only for the high-risk class.

In the univariate analysis, the HCC recurrence was associated with the value of AFP at the time of LT, the diameter of the greatest nodule, the sum of the nodules’ diameters, the presence of MVI on the native livers, the tumoral grade and the RETREAT scores. In the multivariate analysis, only the RETREAT score remained positively associated with the HCC recurrence. The details of the Cox regression analysis for variables associated with recurrence-free survival are shown in Table 3.

**Figure 2:**
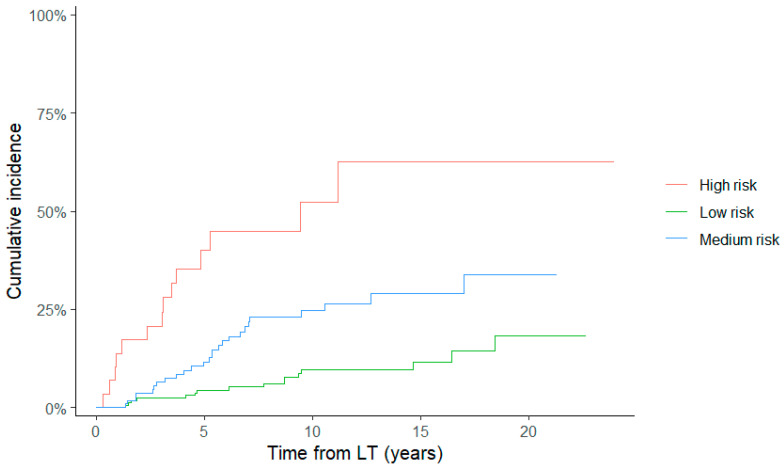
Competing risk analysis: incidence of HCC according to the categories of RETREAT score.

**Figure 3:**
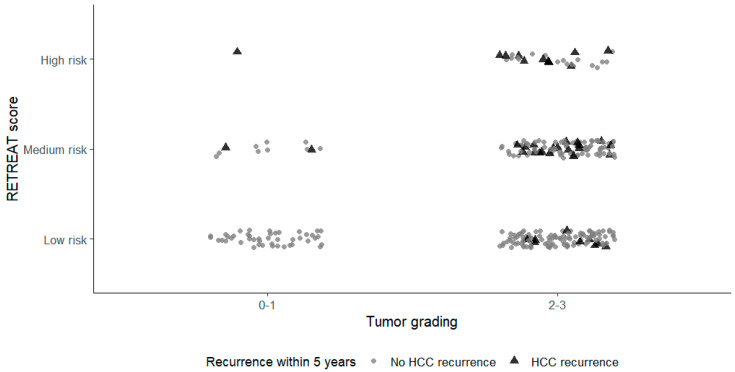
Distribution of patients according to RETREAT score and tumoral grading.

As most patients experienced HCC relapses within 5 years of the LT, we also performed a Cox regression analysis for recurrence within that timeframe. The results were aligned with those observed for the entire follow-up; the multivariate analysis confirmed the unique association of the RETREAT score with the tumoral relapse (Supplementary Table S1). As the observation of HCC recurrence after LT may be prevented by death from other causes, a competitive risks analysis was conducted (Figure 2). In the multivariate analysis for competing risks, the HCC recurrence remained positively associated with the RETREAT score, with an HR of 2.3 for the medium-risk class and 6.4 for the high-risk class compared with the low-risk group (Table 4). A significant correlation between tumor grading and HCC recurrence was observed in the univariate analysis, which was not maintained in the multivariate analysis (Table 3). A scatter plot in Figure 3 depicts the distribution of patients according to the RETREAT score and tumor grading. To assess the prognostic utility of tumor grading for HCC recurrence, we created a modified score, called RETREAT+, where 1 additional point was added to the original RETREAT score in case of tumor grade 3. Although the new model showed a significant association with HCC recurrence in the multivariate analysis, with an HR of 1.55, its predictivity aligned with that of the classic RETREAT score (Supplementary Table S2).

### 3.4. Patient Survival

**Figure 4:**
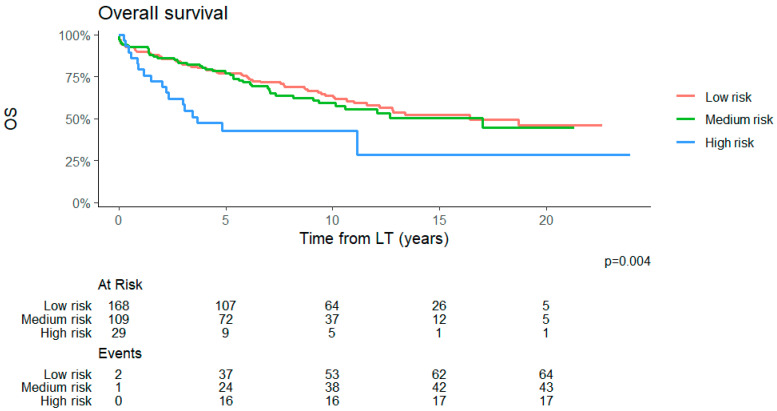
Survival analysis of patients according to the categories of RETREAT score.

During follow-up, we observed 119 deaths: 32 (27%) due to HCC recurrence, 20 (17%) related to other de novo tumors, 19 (16%) infection-related, 28 (24%) due to liver disease recurrence, and 16 (13%) cardiovascular events; the remaining four (3%) occurred due to other causes. The overall survival for the entire population at 1 and 5 years was, respectively, 90% and 74%, with a median overall survival of 13 years. For each RETREAT score category, the 1- and 5-year overall survival was respectively 99% and 95% for the low-risk group, 96% and 86% for the medium-risk group, and 82% and 61% for the high-risk group (Figure 4). The RETREAT score was associated with overall survival in the multivariate analysis only for the highest risk class, which yielded a 2.5 HR of death compared with the lowest risk category (Supplementary Table S3).

## 4. Discussion

Despite the application of more stringent tumoral criteria to select patients for LT, HCC recurrence remains an important issue [22]. Moreover, the recent progressive expansion of LT HCC eligibility criteria, coupled with an increase in down-staging strategies, has fundamentally changed candidate selection, increasing heterogeneity in tumor burden and biological behavior among transplanted patients, leaving, however, post-LT HCC recurrence the major unresolved challenge. In this context, having tools to better select patients to be considered for LT, and/or to manage them once they have undergone LT, becomes fundamental.

MVI is a key pathological factor influencing recurrence and long-term outcomes in HCC, with prognostic heterogeneity based on its presence, extent, and distribution [8]. Radiomics-based approaches for MVI prediction, while promising, currently lack sufficient accuracy and standardization for routine clinical use [9]. Prediction models integrating tumor burden, biological markers, and explant pathology have, therefore, become increasingly important. Among these, the RETREAT score represents the most robustly validated tool. By combining AFP at transplantation, MVI, and explant tumor burden, which are routinely collected in standard transplant practice, RETREAT provides a continuous risk stratification of post-LT tumor recurrence, integrating both biological and pathological determinants of tumor aggressiveness, overcoming the limitations of imaging criteria that do not always accurately predict long-term oncological outcomes.

Meanwhile, the RETREAT score is easy to calculate and is reproducible and readily implementable without the need for complex imaging software or advanced molecular testing. Since its introduction in 2017, the RETREAT score has been widely accepted in Europe and North America as one of the most useful tools in predicting HCC recurrence after LT. The original derivation of the score was based on a large, multicenter cohort from high-volume transplant centers, ensuring adequate statistical power and broad clinical representation [17]. Subsequent validations using national registries and independent international cohorts have consistently demonstrated good discrimination and calibration, supporting the generalizability of the model across different geographic regions and allocation systems, and expanded listing policies [18,19,20,21,22]. Multiple studies have shown that RETREAT outperforms traditional size- and number-based systems in predicting post-LT recurrence, particularly by identifying a subset of patients with very low risk and another with exceptionally high risk of recurrence. This stratification has relevant clinical implications, including the potential to tailor post-LT surveillance intensity and to identify candidates for future adjuvant strategies.

The RETREAT score has demonstrated prognostic value even in patients beyond MC or those who have been successfully down-staged, supporting the concept that tumor biology outweighs static morphologic thresholds in determining outcome. This is particularly relevant nowadays as expanded criteria inevitably include tumors with a wider spectrum of aggressiveness that may increase the risk of futile transplantation in the absence of reliable risk stratification. In fact, beyond candidate selection, prediction scores play a critical role in individualizing post-LT surveillance and management; accordingly, RETREAT-based risk stratification has been proposed to guide the intensity and duration of follow-up imaging and to provide a rational framework for future adjuvant or immunosuppression-modifying strategies. Recent data further suggest that incorporating additional biomarkers such as AFP-L3 and des-gamma-carboxyprothrombin may enhance the prognostic performance of RETREAT, reflecting ongoing efforts to refine biologically driven models [23].

Our study corroborates the ability of the RETREAT score to predict the risk of HCC recurrence after LT for the first time in an Italian setting. This study confirmed the role of the RETREAT score as the main tumoral factor independently associated with HCC recurrence, showing a significant difference in RFS among the three RETREAT risk classes and defining a group (score ≥ 4) at very high risk in which almost half of the patients experienced HCC recurrence within 5 years from LT. Notably, in our study, a RETREAT score of ≥4 was used to define a very-high-risk group based on the observed recurrence distribution within our cohort, rather than modifying the original RETREAT score. Patients with scores of 4, 5, and 6 were grouped together due to the small number of cases with scores of 5 and 6, which would have limited statistical power. This categorization aimed to facilitate clinical interpretation of recurrence risk and is consistent with previous validation studies that applied cohort-specific cut-offs while preserving the score’s continuous nature. In our cohort, patients with RETREAT scores of ≥4 had a markedly increased risk of recurrence, with nearly half experiencing HCC recurrence within 5 years post-transplant, supporting the identification of this subgroup as very high-risk. This approach aligns with prior reports [18], showing that RETREAT scores of ≥4–5 are associated with disproportionately high recurrence rates and allows for clinically meaningful risk stratification without altering the original scoring system.

An important addition of our study to the existing knowledge was the adoption of a competing risks analysis; its use is particularly appropriate in this context, since death from other non-tumoral causes represents an event that precludes the observation of HCC recurrence. The categorization across RETREAT scores remained a significant risk factor for HCC recurrence even when death as a competing event was excluded: the medium- and high-risk RETREAT categories showed, respectively, a 2.3- and 6.4-fold higher risk of HCC recurrence after LT compared with lower RETREAT scores.

As observed by Mehta et al., we also found that the tumoral grading was significantly correlated with HCC recurrence in the univariate analysis, but lost its influence in the multivariate analysis. Nevertheless, the scatter plot in Figure 3 shows that HCC recurrence within 5 years occurred mainly in higher tumoral grades (2/3), while in lower grades, recurrence was observed only when the RETREAT scores were ≥2. We evaluated a new predictive model that integrated tumoral grading and the RETREAT score (RETREAT+). This model was as effective as the original RETREAT score in predicting HCC recurrence, indicating that the addition of tumoral grading did not significantly improve predictive performance. This finding supports the parsimony and clinical adequacy of the original RETREAT model, while also highlighting the need for future refinement using pre-transplant features or molecular biomarkers rather than additional explant-derived variables.

Despite the lack of independent statistical significance of tumoral grading, our results further support the concept that tumor biological behavior is of paramount importance and should be integrated with morphological parameters.

In the last decade, indication to LT has driven a shift from benign to oncological indications [24], reinforcing the need for prognostic tools capable of assessing individual recurrence risk after LT and identifying recipients who may benefit from intensified surveillance, adjuvant therapies, or future immunotherapeutic strategies when validated protocols become available. Nowadays, several AI-based models have been created to predict the risk of HCC recurrence after LT, and they generally outperform the traditional statistical methods [25,26]. However, direct and simple scores still represent an attractive tool for prompt discrimination of patients who are at significant risk of tumor relapse.

Our study provides several important contributions to the existing literature on the use of the RETREAT score. First, unlike most previously published validation studies, which were conducted over relatively short and homogeneous timeframes, our analysis spans more than two decades of liver transplantation activity (2000–2022) within a single high-volume European center. This extended observation period allowed us to capture the progressive evolution of transplant eligibility criteria, including the transition from the Milan Criteria to expanded ones (Up-to-Seven) and the increasing use of LRTs as down-staging strategies. As such, our cohort reflected real-world changes in transplant practice that were not fully represented in earlier European validation studies. Second, our study evaluated the performance of the RETREAT score in a heterogeneous population transplanted under both restrictive and expanded eligibility policies. This aspect is particularly relevant in the contemporary transplant oncology landscape, where extended criteria and down-staging have become standard practice. Our results demonstrated that RETREAT maintains robust prognostic performance across this heterogeneous setting, supporting its generalizability beyond the original derivation cohorts. Third, we adopted a competing risk analysis to account for death without recurrence, which represents a clinically relevant event that precludes the observation of HCC recurrence and has been inconsistently addressed in prior studies. Even after accounting for competing mortality, RETREAT remained independently associated with recurrence risk, further strengthening the reliability of our findings. Finally, our study provides a long-term assessment of recurrence risk, with follow-up extending up to 15 years after liver transplantation. While most previous studies focused primarily on 5-year outcomes, our data confirm that RETREAT is informative not only for early recurrence, but also for guiding long-term surveillance strategies. Importantly, the stability of RETREAT performance across a period marked by major changes in transplant practice suggests that the score captures fundamental aspects of tumor biology that are less influenced by temporal variations in management strategies.

Despite these strengths, we also recognized some limitations intrinsically linked to the need for explant pathology, which makes the score not applicable in the pre-LT setting, limiting its utility for upfront candidate selection or organ allocation decisions. Moreover, in a study that extended over more than two decades, significant changes occurred in locoregional therapies, imaging techniques, post-transplant management, and transplant eligibility criteria, all of which may potentially influence recurrence risk. However, this temporal heterogeneity represents a strength rather than a limitation of our study, as it reflects the real-world evolution of transplant oncology practice. One of the primary aims of our work was to assess the robustness of the RETREAT score across different eras of liver transplantation, characterized by progressively expanding listing criteria and increasing use of down-staging strategies. Our findings demonstrate that RETREAT maintains strong prognostic performance despite these changes, supporting its applicability across evolving clinical contexts.

The monocentric design of this study mitigates some of the variability associated with temporal heterogeneity, as listing policies, locoregional treatment protocols, immunosuppressive regimens, and post-transplant surveillance strategies followed consistent institutional frameworks over time, even as they evolved. We acknowledge that subgroup or sensitivity analyses stratified by transplant era could further explore the impact of temporal changes. However, the relatively low number of recurrence events and the uneven distribution of patients across RETREAT risk categories limited the statistical power required for reliable era-specific analyses.

Finally, another limitation concerns the assessment of MVI. In our cohort, MVI was recorded as a dichotomous variable (present/absent) based on the original pathological reports available at the time of liver transplantation. Although recent studies have demonstrated that refined histopathological grading of MVI—taking into account the number of invaded microvessels and their spatial distribution relative to the tumor—provides additional prognostic information in HCC, a retrospective re-evaluation of all explanted liver specimens according to these contemporary classification systems was not feasible in the current study. Importantly, the primary objective of our work was to validate the prognostic performance of the RETREAT score in a real-world clinical setting. As MVI is incorporated into the RETREAT score as a binary variable, our approach is consistent with the original score definition and reflects routine pathological reporting in clinical practice during the study period. Nevertheless, future prospective studies adopting standardized and detailed MVI grading may clarify whether integrating refined pathological features of MVI into existing prognostic models, including RETREAT, could further improve post-transplant recurrence risk stratification in patients with hepatocellular carcinoma.

## 5. Conclusions

Our study confirms that the RETREAT score is a simple and reliable tool to stratify patients according to post-transplant HCC recurrence risk. In particular, patients classified as high-risk (RETREAT ≥ 4) face a markedly increased likelihood of recurrence, underscoring the need for close post-LT surveillance, as the predicted recurrence risk is over 50%. While this highlights the potential utility of future adjuvant treatment strategies and more intensive monitoring for high-risk individuals, such proposals must be considered hypothesis-generating, given the current lack of interventional evidence. Prospective studies are warranted to determine whether RETREAT-guided interventions can improve long-term outcomes in this population.

## Figures and Tables

**Table 1:** Characteristics of the enrolled patients at time of liver transplantation.

Characteristic	N = 298
Age, year, median (IQR)	58 (53–63)
BMI, kg/m^2^, median (IQR)	25.6 (23.3–28.0)
Male, *n* (%)	267 (89)
Etiology, *n* (%)	
Alcohol	62 (21)
Hepatitis B virus	71 (24)
Hepatitis C virus	135 (45)
Other etiology	30 (10)
Waitlist time, months, median (IQR)	4 (2–7)
MELD score, median (IQR)	11 (8–16)
Bilirubin, mg/dL, median (IQR)	1.60 (1.00–2.80)
INR, ratio, median (IQR)	1.30 (1.16–1.50)
Creatinine, mg/dL, median (IQR)	0.78 (0.68–0.93)
AFP, ng/mL, median (IQR)	9 (4–28)
Child–Pugh classes °, *n* (%)	
A	112 (42)
B	107 (40)
C	50 (18)
Number of HCC treatments, *n* (%)	
0	43 (14)
1	122 (41)
2	75 (25)
3	49 (17)
≥4	9 (3)

° In 269 cirrhotic patients; BMI: body mass index; MELD: Mayo End-Stage Liver Disease; AFP: alfa fetoprotein.

**Table 2:** HCC treatments and RETREAT score at liver transplantation.

Characteristic	N = 298
Number of nodules (explant histology), *n* (%)	
Monofocal	133 (44)
Bifocal	59 (20)
Multifocal	106 (36)
Largest nodule size (cm, histology),	
median (Q1, Q3)	2.5 (2.0, 3.5)
Sum of diameters (cm, histology),	
median (Q1, Q3)	4.0 (2.7, 5.5)
Up-to-Seven criteria (histology), *n* (%)	259 (87)
Milan Criteria (histology), *n* (%)	198 (66)
Vascular invasion, *n* (%)	65 (22)
Tumor grade, *n* (%)	
0	48 (16)
1	9 (3)
2	126 (42)
3	115 (39)
RETREAT score, *n* (%)	
0	4 (1)
1	157 (53)
2	52 (17)
3	56 (19)
4	16 (5)
5	9 (3)
6	4 (1)
RETREAT score (category), *n* (%)	
Low risk (0–1)	161 (54%)
Medium risk (2–3)	108 (36%)
High risk (4–6)	29 (10%)

**Table 3:** Cox model for HCC recurrence.

	Univariate		Multivariable	
Variable	HR (95% CI)	*p*-Value	HR (95% CI)	*p*-Value
Age	1.00 (0.96 to 1.04)	0.82	1.00 (0.96 to 1.04)	0.96
Etiology		0.020		
Alcohol	—		—	
Hepatitis B virus	0.62 (0.25 to 1.52)		0.56 (0.22 to 1.39)	0.21
Hepatitis C virus	1.46 (0.72 to 2.98)		1.05 (0.51 to 2.18)	0.89
Other etiology	0.41 (0.11 to 1.49)		0.41 (0.11 to 1.50)	0.18
Tumor grade (3)		0.002		
0–1	—		—	
2–3	4.27 (1.33 to 13.7)		2.56 (0.78 to 8.38)	0.12
RETREAT score (category)		<0.001		
Low risk	—		—	
Medium risk	2.74 (1.44 to 5.20)		2.34 (1.22 to 4.52)	0.011
High risk	8.57 (4.10 to 17.9)		6.67 (3.12 to 14.3)	<0.001

CI = confidence interval; HR = hazard ratio with 95% CI.

**Table 4:** Fine–Gray model for competing risk analysis (HCC recurrence).

Variable	sHR (95% CI)	*p*-Value
Age	1.0 (0.97 to 1.1)	0.560
Etiology		
Alcohol	—	
Hepatitis B virus	0.65 (0.26 to 1.6)	0.340
Hepatitis C virus	1.2 (0.57 to 2.3)	0.690
Other etiology	0.45 (0.13 to 1.6)	0.210
Tumor grade (3)		
0–1	—	
2–3	2.7 (0.76 to 9.3)	0.130
RETREAT score (category)		
Low risk	—	
Medium risk	2.3 (1.2 to 4.5)	0.017
High risk	6.4 (2.9 to 14)	<0.001

CI = confidence interval; HR = hazard ratio values are subdistribution hazard ratios (sHRs) with 95% CI; *p*-values from Wald tests.

## Data Availability

The data from the present study are kept confidential but can be provided upon reasonable request to the authors.

## Supplementary Materials

The following supporting information can be downloaded at: https://www.mdpi.com/article/10.3390/cancers18040556/s1, Table S1: Overall survival at 5 years; Table S2: RETREAT vs RETREAT+; Table S3: Cox model for overall survival.
