# Supplementary material for: RETREAT Score Accurately Predicts the Long-Term Risk of HCC Recurrence After Liver Transplantation: A Single-Center Real-Life Validation
Source: Cancers (Basel). 2026 Feb 9;18(4):556. doi: 10.3390/cancers18040556 (PMC12938944; doi:10.3390/cancers18040556)
Supplement: Supplementary file 1 [file cancers-18-00556-s001.zip › cancers-4130393-supplementary.pdf]

Supplementary Table 1: Overall survival at 5 years

	Univariate		Multivariate	
**Variables**	**HR (95% CI)**	**p-value**	**HR (95% CI)**	**p-value**
Age	1.00 (0.95 to 1.04)	0.87	1.00 (0.96 to 1.05)	0.85
Etiology		**0.012**		0.080
Alcohol	—		—	
Hepatitis B Virus	0.82 (0.29 to 2.34)		0.76 (0.27 to 2.18)	
Hepatitis C Virus	1.78 (0.77 to 4.10)		1.31 (0.56 to 3.07)	
Other etiology	0.21 (0.03 to 1.68)		0.20 (0.03 to 1.67)	
Tumor grade (3)		**0.034**		0.38
0-1	—		—	
2-3	2.96 (0.91 to 9.59)		1.66 (0.50 to 5.52)	
RETREAT score (category)		**<0.001**		**<0.001**
Low risk	—		—	
Medium risk	3.61 (1.65 to 7.88)		3.16 (1.43 to 7.01)	
High risk	9.31 (3.85 to 22.5)		7.24 (2.91 to 18.0)	

CI = Confidence Interval, HR = Hazard Ratio Values are subdistribution hazard ratios (sHR) with 95% CI; p-values from Wald tests.

Supplementary Table 2: RETREAT vs RETREAT+

	RETREAT model		RETREAT + Grading model	
**Variable**	**HR (95% CI)**	**p-value**	**HR (95% CI)**	**p-value**
**RETREAT score**	1.6 (1.4 to 1.9)	**<0.001**		
**RETREAT score**			1.5 (1.3 to 1.8)	**<0.001**

CI = Confidence Interval, HR = Hazard Ratio. Values are subdistribution hazard ratios (sHR) with 95% CI; p-values from Wald tests

Supplementary Table 3: Cox model for overall survival

	Univariate		Multivariate	
**Features**	**HR (95% CI)**	**p-value**	**HR (95% CI)**	**p-value**
Age	1.01 (0.98 to 1.03)	0.64	1.01 (0.98 to 1.04)	0.47
Etiology		0.15		
Alcohol	—		—	
Hepatitis B Virus	0.60 (0.35 to 1.05)		0.58 (0.33 to 1.01)	0.054
Hepatitis C Virus	0.86 (0.54 to 1.37)		0.81 (0.51 to 1.30)	0.38
Other etiology	0.55 (0.27 to 1.11)		0.55 (0.27 to 1.12)	0.10
Tumor grade (3)		0.81		
0-1	—		—	
2-3	1.06 (0.67 to 1.68)		0.87 (0.54 to 1.41)	0.57
RETREAT score (category)		0.011		
Low risk	—		—	
Medium risk	1.13 (0.76 to 1.68)		1.12 (0.75 to 1.69)	0.58
High risk	2.49 (1.44 to 4.29)		2.54 (1.44 to 4.47)	0.001

CI = Confidence Interval, HR = Hazard Ratio with 95% CI.
